# A Serial Mediation Model of the Relationship between Cybervictimization and Cyberaggression: The Role of Stress and Unforgiveness Motivations

**DOI:** 10.3390/ijerph17217966

**Published:** 2020-10-29

**Authors:** Cirenia Quintana-Orts, Lourdes Rey, María Teresa Chamizo-Nieto, Everett L. Worthington

**Affiliations:** 1 Department of Personality, Evaluation and Psychological Treatment at the University of Granada, 51001 Campus Ceuta, Spain; 2 Department of Personality, Evaluation and Psychological Treatment at the University of Malaga, 29071 Malaga, Spain; lrey@uma.es (L.R.); mtchamizo@uma.es (M.T.C.-N.); 3 Department of Psychology, Virginia Commonwealth University, Richmond, VA 23284, USA; eworth@vcu.edu

**Keywords:** cyberbullying, aggression, victimization, stress, unforgiveness motivations, stress-and-coping

## Abstract

Cyberaggression is often triggered by cybervictimization. However, little attention has been given to the underlying mechanisms in this relationship. Specifically, this study examined the mediating roles of stress as well as unforgiveness (i.e., revenge and avoidance motivations) in the cybervictimization-cyberbullying aggression link. The main goal is to investigate the direct and indirect effects of cybervictimization on cyberbullying aggression while modeling a process in which cybervictimization causes stress, which in turn causes unforgiveness motivations concluding with cyberbullying aggression as the consequent. A total of 979 adolescents (*M*_age_ = 13.72, *SD* = 1.31) completed the relevant scales at two time points spaced four months apart. The results confirm that stress and revenge motivation at Time 1 act as serial mediators between cybervictimization at Time 1 and cyberbullying behaviors at Time 2. Additionally, the results reveal that avoidance at Time 1 was not a significant mediator in the links between cybervictimization at Time 1 and cyberbullying aggression at Time 2. Our findings provide support for the stress-and-coping model of forgiveness in adolescence and offer original insight into the developmental process of bully-victims in cyberbullying context. These results suggest the importance of efforts addressing motivations and emotion-focused coping strategies in adolescents who have been bullied to prevent and reduce those adolescents’ future stress and aggressive behaviors. The contributions and implications of the results are discussed.

## 1. Introduction

Cyberbullying is a prevalent transgression in many adolescents’ everyday life around the world [1,2]. It refers to repeated acts of harassment, characterized by an imbalance of power between the aggressor and victim that includes threats or exclusion through social networks, as well as electronic and communication technologies to a victim who has difficulties defending themselves [3,4]. Although many studies have focused on either pure cyberbullies or pure cybervictims, relatively few have explored the group composed of those who both are victims of cyberbullying and have cyberbullied others (i.e., cyberbully-victims) and develop serious mental health concerns [5]. Regarding the formation of cyberbully-victims, research that examines the associations and underlying mechanisms between victimization and aggression is still limited [2,6]. Previous studies suggest that one of the strongest predictors of engaging in cyberbullying aggression is being a previous victim of cyberbullying [2,5,7,8]. To date, few studies have examined the mechanisms underlying why cybervictims attack others and then become cyberbully-victims. Identifying mechanisms through which cybervictimization is related to cyberbullying aggression has significant implications for the development of tailored and effective interventions aimed at reducing the negative impacts of cyberbullying victimization. The present study aims to fill in this gap.

### 1.1. The Mediating Role of Stress

Drawing on Worthington’s model [9] and Lazarus and Folkman’s [10] stress-and-coping model, transgressions are interpersonal stressors that may lead to different appraisals (e.g., avoidance or revenge) that depends on the personal perceptions of the interpersonal threat and the resources available to cope with the offense. As a result, the interpersonal stressors create different physiological, cognitive, motivational, behavioral, and emotional stress reactions [9,11]. Although the majority of prior literature focused on adults’ interpersonal transgressions, it seems applicable to adolescents who have been (cyber)bullied [9,12].

Within this model, cyberbullying can be understood as the interpersonal transgression that adolescents appraise to be a threat [8,13]. Previous work shows peer victimization is associated with significantly more stress, perceived and measured by physiological markers of stress such as cortisol [8,14,15,16]. Specifically, those individuals who experience cyberbullying have been found to report higher stress symptoms (e.g., headaches or cortisol secretion) [14,15,17]. The unawareness of cybervictims of who is cyberbullying them would likely exacerbate stress, adversely influencing their mental health and regulation processes (e.g., [8,18,19]).

Empirical work has shown that stress is linked to higher risk of becoming perpetrators of bullying [16,20]. In an effort to alleviate this internal negative affective state, different appraisal strategies may lead individuals to engaging in particular responses to the situation: some individuals might do nothing, others might flee or avoid the situation or might call for help, whereas others might react aggressively toward another person by engaging in cyberbullying aggression in response to victimization [7,8,21]. Thus, how the strain of cybervictimization is appraised is an important factor that influences their reactions to that stress and, relatedly, their responses and coping strategies.

### 1.2. The Mediating Role of Unforgiveness Motivations

One of the many ways individuals cope with a stressor is by the emotion-focused coping strategies [11,13]. The experience of cybervictimization, as a repeated, chronic, and uncontrollable stressor, can evoke victims’ strong negative emotions (e.g., angry, frustration) [21,22], which leads to different stress reactions such as unforgiveness motivations [9,15,23]. Within the emotion-focused coping strategies, individuals can try to alleviate the levels of stress by doing nothing (avoidance motivation) or by bullying back in order to exact revenge (revenge motivation) [7,12,24].

Revenge motivations have been found to significantly predict whether a victim of bullying and/or cyberbullying turns into a bully, choosing a prior offender as the target for cyberbullying or a third person [7,25,26]. Revenge is associated with an increase in stress (e.g., [27]) and greater aggressiveness, anger, and hostility [25,28]. A previous study found a mediational effect of revenge motivation in the relationship between victimization and aggression in traditional bullying [26]. León-Moreno et al. [26] suggest that victimized adolescents would express violent behaviors at schools as the result of the desire to take revenge on their aggressor, so they probably assault those who assaulted them. It plausible that, when cybervictimization occurs, increased perceptions of the encounter as stressful lead some adolescents to deal with such situations in a vengeful way such as sending a cyberbullying message back to the transgressor [8,16,21].

With regard to avoidance motivation, some victims of cyberbullying and bullying are likely to engage in avoidance motivations and behaviors [24,29,30]. This motivation may seem less directly related to violence as it includes the victim’s attempts to mentally or physically disengage from the stressful situation [29,31]. León-Moreno et al. [26] found no mediational effect of avoidance motivation between victimization and aggression in traditional bullying context. By contrast, some studies pointed out that this motivation can also constitute a risk factor. Although avoidance may restrain negative emotions in the short term [25], it can hamper the resolution of the transgression over time by amplifying stress (e.g., [24]) and unwanted thoughts and emotions (e.g., [25,32]). Thus, it is possible that after uncontrollable stress resulting from cybervictimization, some adolescents develop a response style of unwillingness to continue being subjected to it (i.e., avoidance) and, over time, this would increase aggressive behaviors in order to resolve ambiguous or harmful interactions with their peers [24,32].

### 1.3. A Serial Multiple Mediation Model

There is a scarcity of empirical research on the association between cybervictimization and cyberbullying aggression. Some authors suggest that strain factors, such as stress and unforgiveness motivations, may explain why people reporting higher cybervictimization might increase the likelihood of becoming a bully [21,26]. Although not yet tested, it is reasonable to speculate that stress and unforgiveness motivations (i.e., revenge and avoidance) could play mediational roles. To begin filling the gap in the current lack of knowledge on the association between cybervictimization and cyberbullying aggression, the current study aims to analyze how stress and unforgiveness motivations work together as two mediators in a serial mediation model in a sample of adolescents over a period of four months.

**Figure 1:**
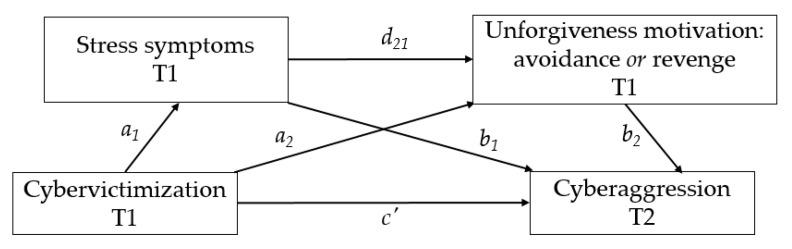
Proposed serial mediation model linking cybervictimization to cyberbullying aggression through stress and unforgiveness motivations (i.e., avoidance or revenge) as serial mediators. Note: *a*, *b*, *c*, *c*′, and *d* represent path coefficients.

The serial multiple mediation model (Figure 1), compared with a simple mediation model, allows for simultaneous examination of multiple mechanisms from the antecedent variable to the consequent variable in a single integrated model [33] which can offer more insights into how cybervictimization is related to cyberaggression. As such, addressing underlying mechanisms has important implications for the improvement of theory, by helping advance research on the dynamics of bully-victims and contributing on the prevention and intervention of cyberaggression after experiencing cybervictimization.

Grounded in the stress-and-coping model of forgiveness [9,12], we propose that adolescents who suffer cybervictimization are vulnerable to experience increased levels of stress (T1), which may cause them to react and cope negatively in response (unforgiveness motivations: revenge or avoidance) (T1) leading to aggressive behaviors (cyberaggression, T2) (Figure 1).

The present study employed a prospective design to analyze whether stress and unforgiveness motivations (i.e., revenge and avoidance) work together sequentially. Specifically, building upon the aforementioned research, the following research hypotheses are stated:

**Hypothesis** **1.** *Cybervictimization would indirectly heighten risk for cyberaggression; specifically, in serial fashion, greater cybervictimization would be related to higher levels of stress (T1) and revenge motivation (T1), which, in turn, would be related to higher cyberaggression (T2).*

**Hypothesis** **2.** *Cybervictimization would indirectly heighten risk for cyberaggression; specifically, in serial fashion, greater cybervictimization would be linked to higher levels of stress (T1) and avoidance motivation (T1), which, in turn, would be related to higher cyberaggression (T2).*

## 2. Materials and Methods

### 2.1. Participants and Procedures

The initial sample consisted of 1224 adolescents from five education centers of the province of Malaga (south of Spain), who completed a battery of questionnaires in the first time (T1) of this prospective study. From them, 1046 adolescents participated again in the second time (T2). A total of 245 students’ data were eliminated: 67 adolescents did not correctly fill out the batteries of questionnaires or did not report necessary demographic data (i.e., gender and age); and 178 students did not participate again in T2. Therefore, the total sample in the current study consisted of 979 adolescents (543 females) aged 12–18 years (*M*_age_ = 13.72, *SD* = 1.31). Their distribution by academic grade was: 7th (26.3%), 8th (26.9%), 9th (23.2%), and 10th (23.7%). The majority of students were Spanish (97.4%); seven participants did not report their nationality.

The data were collected between January/February (T1) and May/June (T2) of 2019. The procedure was to administrate a battery of questionnaire to adolescents anonymously and voluntarily during one hour in a tutorial class at both Time 1 and Time 2. The Declaration of Helsinki [34] was followed, and we obtained the permission from the Ethical Committee of University of Malaga. Twelve education centers were contacted to participate in the research. Five of them participated. Both directors and students’ parents were informed of the methodology of the research. In four centers, the parents signed a consent form so that the adolescents could participate in the study. In one center, the adolescents, whose parents did not refuse their participation, completed the batteries of questionnaires. During the administration of questionnaires, two researchers and one teacher were presented. Firstly, the researchers reminded students of their anonymity and voluntary participation, noting that they could withdraw the study at any point freely and without detriment. Later, some verbal instructions were given, and any question about the completion of the battery of questionnaires was resolved.

### 2.2. Measures

The battery of questionnaires included some questions about demographic information (i.e., sex, age, nationality, and grade).

To assess the cyber-bullying, the European Cyberbullying Intervention Project Questionnaire was used (ECIP-Q) [35]. This questionnaire consisted of 22 items that assess the frequencies of suffering cybervictimization (11 items) and engaging in cyberbullying aggression (11 items) during the last two months. Adolescents answered this questionnaire throughout five-point response options ranging from 0 = Never to 4 = More than one once a week. The Spanish version, which possessed good psychometric properties, was used in this study [36] with Cronbach’s alpha values of 0.83 and 0.90 obtained for cybervictimization (T1) and cyberaggression (T2), respectively.

Stress was measured by the subscale of stress from Depression, Anxiety and Stress Scales (DASS-21) [37]. This subscale assesses, using 7 items, the frequency of symptomatology of stress, which happened to every participant during the last week. It uses four-point response options, being 0 = Did not apply to me at all, and 3 = Applied to me very much or most of the time. We used the Spanish version with appropriate indexes of estimated reliability [38]. In this study, Cronbach’s alpha value was 0.83.

Transgression-related Interpersonal Motivations-18 Scale (TRIM-18) [39] assesses three motivations (avoidance, revenge and benevolence) toward the person who hurt him or her. In this study, we used the avoidance (7 items) and revenge (5 items) subscales, which are answered using five-point response options from 1 = Strongly disagree to 5 = Strongly agree. In the current study, the Spanish version [40] was utilized, which obtained adequate psychometric properties: Cronbach’s alpha values of 0.85 and 0.87 were obtained for avoidance and revenge subscales, respectively.

### 2.3. Data Analyses

First, all the missing item values were imputed using the expectation-maximization (EM) imputation algorithm with SPSS (v23, IBM, Armonk, NY, USA). Second, SPSS was used to obtain descriptive statistics and correlation analyses. Later, a serial multiple mediation model (Figure 1) was conducted with stress, and unforgiveness motivation (revenge or avoidance) as mediators. To perform serial multivariable mediation analyses, the PROCESS macro (Model 6) [33] was utilized. In serial mediation, mediators (stress and unforgiveness motivation) are expected to have a direct influence on each other [33], and the independent variable (cybervictimization) is assumed to effect mediators in a serial pattern, which then, in turn, influence the dependent variable (cyberaggression). To perform the inference tests for the indirect effects and avoid the effects of heteroscedasticity and non-normality, a heteroscedasticity-consistent standard error (HC3) estimator [41] and 95% bias corrected confidence interval based on a 10,000-bootstrap sample were used [33]. Data were analyzed with age and sex as covariates.

## 3. Results

### 3.1. Descriptive Statistics and Correlation Analysis

Mean and standard deviation, Cronbach’s α, and Pearson correlations among the study variables are shown in Table 1. As expected, the variables showed positive and significant correlations among them.

### 3.2. Serial Multivariable Mediation Analyses

A serial multiple mediation model using Hayes’s SPSS macro PROCESS [33] was conducted (Model 6, Figure 1). The statistical diagram in Figure 1 depicts the serial mediator model in which the variable cybervictimization is modelled as affecting cyberbullying aggression through four pathways (i.e., a_1_b_1_, a_2_b_2_, a_1_d_21_b_2_, c′). Arrows in the figure display the paths of the tested model, and a_1_, a_2_, b_1_, b_2_, d_21_, c, c′, indicate the unstandardized path coefficients.

In this serial mediation, mediators are assumed to have a direct effect on each other [33], and the independent variable (i.e., cybervictimization) is assumed to influence mediators in a serial fashion which then, subsequently, influence the dependent variable (i.e., cyberaggression). Considering the two-mediator model in Figure 1, there are four indirect effects estimated as products of regression coefficients. The indirect effects are considered statistically significant if the 95% bias corrected confidence interval (CI) does not contain zero. To overcome effects of heteroscedasticity and non-normality, heteroscedasticity-consistent standard error (HC3) estimator [41], and a 10,000-bootstrap sample were used [33]. Age and sex were used as covariates in all analyses.

**Figure 2:**
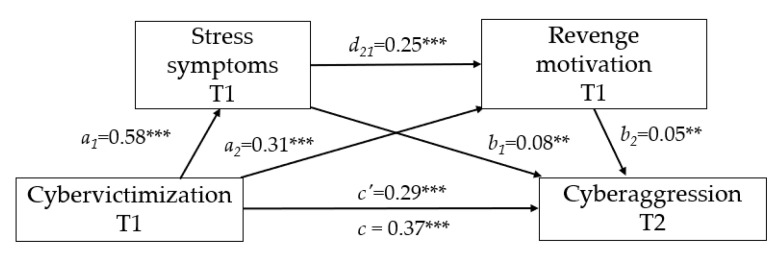
Results of serial multiple mediating test (Model 6) for stress and revenge as mediators. Coefficients are unstandardized; ** *p* < 0.01; *** *p* < 0.001.

Revenge motivation. The results (see Figure 2) show that cybervictimization significantly predicted stress (a_1_ = 0.58; BCa 95% CI = 0.45, 0.71) and revenge motivation (b_1_ = 0.31; BCa 95% CI = 0.14, 0.49). Both stress and revenge had a significant impact on cyberbullying aggression: b_1_ = 0.08 (BCa 95% CI = 0.03, 0.14) for stress; b_2_ = 0.05 (BCa 95% CI = 0.02, 0.09) for revenge.

Figure 2 and Table 2 show that the total effect of cybervictimization on cyberbullying aggression was significant (c = 0.37; BCa 95% CI = 0.24, 0.49). The direct path coefficient between the two variables (c’ = 0.29; BCa 95% CI = 0.16, 0.43) continued to emerge even after when stress and revenge were added to the analysis as serial mediators. Specifically, the 95% bias-corrected confidence interval produced by the PROCESS macro, based on the 10,000 bootstrap method, confirms that the specific indirect effect of cybervictimization on cyberbullying aggression through only stress (T1) (i.e., a_1_b_1_ = 0.05; SE(HC3) = 0.02; BCa 95% CI = 0.02, 0.09) and revenge (T1) (i.e., a_2_b_2_ = 0.02; SE(HC3) = 0.01; BCa 95% CI = 0.01, 0.03) are significant. Lastly, the specific indirect effect of cybervictimization on cyberbullying aggression through the two mediators in serial was confirmed (i.e., cybervictimization (T1) → stress (T1) → revenge (T1) → cyberbullying aggression (T2) (a_1_d_21_b_2_ = 0.01; SE(HC3) = 0.00; BCa 95% CI = 0.00, 0.02). The hypothetical mediating effects were supported (Hypothesis 1).

**Figure 3:**
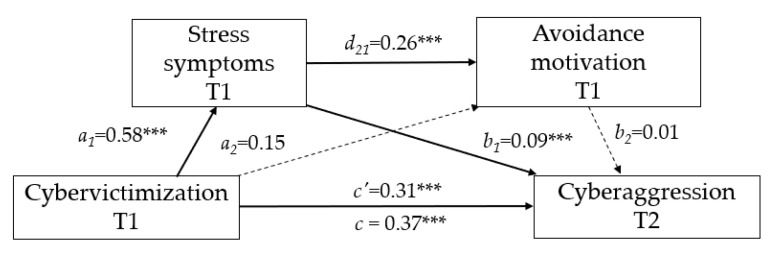
Results of serial multiple mediating test (Model 6) for stress and avoidance as mediators. Coefficients are unstandardized. The dotted lines indicate nonsignificant pathways. *** *p* < 0.001.

Avoidance motivation. The results (see Figure 3 and Table 3) reveal that cybervictimization significantly predicted stress (a_1_ = 0.58; BCa 95% CI = 0.45, 0.71). Stress was also found to have a positive effect on cyberbullying aggression (b_1_ = 0.09; BCa 95% CI = 0.04, 0.15) and on avoidance (d_21_ = 0.26; BCa 95% CI = 0.16, 0.36). However, cybervictimization did not predict avoidance motivation (a_2_ = 0.15; BCa 95% CI = −0.03, 0.33), and avoidance motivation did not show a significant impact on cyberbullying aggression (b_2_ = 0.01; BCa 95% CI = −0.02, 0.04). The total effect (c) of cybervictimization on cyberbullying aggression was significant (c = 0.37; BCa 95% CI = 0.24, 0.49). The direct path coefficient between the two variables (c’ = 0.31; BCa 95% CI = 0.17, 0.44) continued to emerge even after when stress and avoidance were added to the analysis as serial mediators. However, the only specific indirect effect of cybervictimization on cyberbullying aggression was significant through stress (T1) (i.e., a_1_b_1_ = 0.06; SE(HC3) = 0.02; BCa 95% CI = 0.02, 0.09). No significant serial mediational effect (a_1_d_21_b_2_ = 0.00; SE(HC3) = 0.00; BCa 95% CI = −0.00, 0.01) or indirect effect was observed through avoidance motivation (a_2_b_2_ = 0.00; SE(HC3) = 0.00; BCa 95% CI = −0.00, 0.01). Thus, the hypothetical mediating effect was only supported for stress (Hypothesis 2; see Figure 3).

## 4. Discussion

This research examines the mechanisms underlying the relationship over time between cybervictimization and cyberbullying aggression though whether stress and unforgiveness motivations (i.e., revenge and avoidance) act as serial mediators in this association. The results of the serial mediation analysis reveal that the specific indirect effect of cybervictimization on cyberbullying through both stress and revenge motivation is significantly positive. By contrast, no significant serial mediational effect was observed through both stress and avoidance motivation. The findings of this study highlight that cybervictimization is only related to cyberbullying aggression through revenge motivation, but not through avoidance motivation.

This study also indicates that cybervictimization might increase stress symptoms, which might lead some adolescents to alleviate this internal state by engaging in aggressive response such as cyberbullying aggression. This finding is consistent with previous studies that highlight stress and different stressors, such as being a previous victim, as important aspects of externalizing problems (e.g., delinquency, or cyberbullying) [8,16,21]. Thus, cybervictimization might create different physiological, emotional and motivational stress reactions, leading some adolescents to use poor coping strategies, which might lead to cyberbullying aggression, in “an effort to alleviate the pressure and negative emotions resulting from the strain of cybervictimization” [12,16]. Taken together, our findings partially support the applicability of the adult stress-and-coping model of forgiveness to adolescents [9,12].

### 4.1. Hypothesis 1

Our findings showed cybervictimization as a source of stress that could make adolescents take revenge at Time 1, making victimized adolescents more at risk for cyberbullying aggression at Time 2. This means that adolescents with more frequent cybervictimization experiences have a higher tendency toward stress; this, in turn, results in an increase in revenge motivations at Time 1, and conclude with cyberbullying aggression four months later at Time 2. Our results confirm that cybervictimized adolescents may become involved in online violent behavior, assaulting those who assaulted them or displacing aggression onto others [7,8,21], as a result of increases in both stress and the desire to exact revenge. This result is in line with the stress-and-coping model of forgiveness [9] and previous research (e.g., [8,26]) suggesting that when the interpersonal situation is appraise as a strong threat, adolescents might experience negative emotional reaction and revenge motivation, which may be alleviate by expressing violent behaviors [12,42]. Revenge motivation can be explained by an increase in negative emotions such as resentment, bitterness, anger, fear or hostility [42,43] and rumination [44,45] that can lead to interpret social cues in hostile way [21] and, consequently, this heightens the likelihood of becoming involved in violent behavior such as cyberbullying [46,47]. It is possible that when cyberbullying occurs, as a repeated and chronic offense characterized by imbalance of power between the aggressor and victim, the victims’ revenge motivations operate as a way of restoring the power balance and increasing their sense of security and control [28,48].

There are, of course, other alternative theoretical frameworks within which to view the current findings. For example, in social learning theory [49,50], the victim has seen personally ways to victimize and perhaps seen that the victimizer has not been punished. Thus, the cybervictim not only has those aggressive behaviors in his or her social learning repertoire of behaviors but might find that aggression against others has been disinhibited by the violence or aggression against the self that has been witnessed. Being a victim will affect stress and unforgiveness, both of which could promote a socially learned aggressive response against others. In addition, Bandura’s moral disengagement theory argues that some circumstances can convince a person that moral rules do not apply to oneself in a particular situation. Thus, being a victim of cyberbullying or bullying might persuade the person that he or she has the “moral right” to act similarly, and that feeling of moral justification to inflict hurt on others might be seen as revenge. There are physiological and conditioning theories [51] that say that pain is linked physiologically to aggression. For example, a rat who is in a cage with another rat is foot shocked. That rat will often attack the cage-mate.

### 4.2. Hypothesis 2

Although avoidance motivation was hypothesized as an important serial mediator together with stress in the relationship over time between cybervictimization and cyberbullying aggression, our results reveal no sequentially indirect effect of cybervictimization on cyberbullying aggression through both stress and avoidance. This finding is in accordance with the results found by León-Moreno et al. [26] in traditional bullying contexts. In their study, they did not find a mediational effect of avoidance between victimization and bullying aggression. A possible explanation may rely on the assumption that avoidance motivation may elicit empathy for the bully by decreasing negative affect, cognitive evaluations, and stress in the short term [25,52]. In fact, a combination of short-term avoidance and longer-term forgiveness has been suggested as a possible effective therapeutic intervention strategy for victims of bullying, avoidance being a first step in the forgiveness process [25]. However, there are many gaps in these assumptions and future studies on the impact and relation between avoidance motivation and forgiveness are required [25].

Although avoidance did not show relations with cyberbullying aggression, our results also reveal a noteworthy and positive effect of stress on avoidance motivation, as suggested in the stress-and-coping model [9,11]. In line with previous research [24], victimization is associated with increased stress and avoidance. It may be that this increase in stress and consequent avoidance motivation are more related to passive behavioral responses such as rumination [53] and cognitive distancing [54] instead of aggressive behaviors. However, literature points to poor outcomes for avoidance in the shorter and longer term, such as risk for future victimization [32] or suicidal ideation [15,55]. Thus, further investigation regarding the associations between cybervictimization, stress, avoidance motivations and the impact of passive behavioral responses is required.

### 4.3. Limitations

Although this study presents several strengths, such as the prospective design, several limitations should be acknowledged. First, the generalizability is limited. On account of convenience sampling, our sample was not representative of the Spanish adolescent population. Future studies should consider different regions of Spain to make the generalization of the results possible. A second limitation is the lack of physiological measures to assess stress levels that may help us to understand the relationship between cyberbullying victimization and cyberbullying aggression such as the skin conductance (e.g., [56]). Future research, using alternative techniques, may wish to delve into how cyberbullying victimization, stress and unforgiveness motivations interact to predict cyberbullying aggression. In addition, daily diary studies would also be helpful to better understand how and when these different processes unfold over time.

As a third limitation, our research did not include other important variables and factors highly correlated with cyberbullying that could have been informative. For example, follow-up research investigating in a more integrated model the combination of unforgiveness motivations, stress and relevant factors such as the perceived severity of cybervictimization [46,48], victims’ negative emotional impact [57] and socioemotional competences [12,58], among others, is necessary. Furthermore, as one of the strongest predictors of cyberbullying aggression is traditional victimization (e.g., [2]), it would be interesting to examine the proposed mediational model as a possible causal relationship that may exist between traditional bullying and cyberbullying aggression.

### 4.4. Implications

Although this study reflected limitations and a need for additional research, the results may contribute to the understanding and possible implications related to the links among adolescents’ involvement in cyberbullying aggression. The results of this study provide support for the cybervictimization (T1) → stress (T1) → revenge (T1) → cyberbullying aggression (T2) causal sequence, as one of the many developmental process of cyberbully-victims [6,59].

Given the prospective positive impact of cybervictimization on adolescents’ stress symptoms and revenge motivations, adults should pay serious attention to cybervictimization of adolescents in efforts to ensure healthy coping strategies to solve conflicts and transgressions, as well as fostering positive peer environments. Educators and families should look for different approaches to help adolescents who suffer from cybervictimization. In practice, this study suggests that assessing and addressing the nature of respondents’ victimizing experiences, including stress and adolescents’ feelings, thoughts, and intentions towards their aggressors may help to identify individuals more at risk of maladaptive coping with victimization and to provide them suitable interventions [16,59]. For example, families may facilitate effective coping strategies, avoiding revenge or avoidance advices to cope with cybervictimization. Moreover, school professionals could directly teach stressed adolescents to identify negative emotions and motivations to develop more adaptive thoughts and coping strategies to deal with interpersonal and stressful events. Making adolescents more conscious about the negative outcomes of avoidant and vengeful coping when adolescents confront difficulties, may improve resolutions in more effective ways and decrease aggressive behaviors such as cyberbullying.

Addressing the motives and processes that contribute to bullying aggression is pointed out as an important part of effective school responses to bullying behavior [59]. One promising emotion-focused coping method that is intended to reduce negative emotions, thoughts, and behaviors after cyberbullying experience is forgiveness. Forgiveness is formalized in the stress-and-coping model of forgiveness in adults [9,60]. Forgiveness is argued to be an effective emotion-focused coping strategy that can reduce the sense of threat appraisal and the level of unforgiving emotions and motivations, such as revenge, in bullying and cyberbullying settings [12,23,25,61]. Previous meta-analysis and systematic reviews highlight forgiveness as a coping mechanism found to have many positive and a few negative effects for adolescents who had been victims of interpersonal transgressions such as bullying [12,62,63]. Thus, further work is needed regarding the interplay between forgiveness and unforgiveness processes related to the developmental process of bully-victims in an online context.

As a public health concern, cyberbullying is also a trigger of poor mental health such as the development of affective disorders, depression, loneliness, suicidal ideation, and psychosomatic symptoms [2,15,19]. Among those involved, cyberbully-victims are the group who presents more deleterious mental health problems [5]. Given that young people spend so much time online, and they suffer the adverse impact of the negative aspects of social networking, prevention and intervention should fall not only on education, but should also include a cross-sectoral response involving health, technology, and legal considerations [64]. As per Spears et al. [64], there is a need to promote available online sources (e.g., by those administrating public health prevention) to positively impact on young people’s way of socializing and well-being. For example, engaging youth and co-creating the online resources with them might be important steps to reach a larger and more diverse number of youths when other sources of help (i.e., parents and peers) are inadequate or unavailable.

## 5. Conclusions

Despite its limitations, as far as we know, this is the first prospective serial follow-up study to examine why cybervictims may attack others becoming cyberbully-victims. The current study took an important step in the applicability of the adult stress-and-coping model of forgiveness [9,13]. Our findings confirm that cybervictimization was positively related to higher stress symptoms, leading some adolescents to develop revenge motivations that triggered cyberbullying aggressions. Thus, higher levels of revenge motivations can represent a risk for cyberbullying aggression after suffering cybervictimization. However, avoidance motivation was not found as a significant mediator to predict cyberbullying aggression. Future studies in the field of cyberbullying should consider individual’s stress symptoms and emotion-focused coping when designing interventions to prevent cyberbullying aggressions.

## Figures and Tables

**Table 1:** Descriptive statistics, estimated internal consistency, and correlations among variables.

	1	2	3	4	5
1. Cybervictimization (T1)	-				
2. Stress (T1)	0.33	-			
3. Avoidance (T1)	0.13	0.24	-		
4. Revenge (T1)	0.17	0.19	0.41	-	
5. Cyberaggression (T2)	0.34	0.25	0.09	0.21	-
Mean	0.23	0.89	3.12	2.18	0.18
Standard deviation	0.38	0.71	1.02	1.02	0.42
α	0.83	0.83	0.85	0.87	0.90

Note: All correlations are significant at *p* < 0.01. T1 = Time 1; T2 = Time 2.

**Table 2:** Regression coefficients, standard errors, and model summary information for stress and revenge as mediators.

	Consequent											
		M _1_ (Stress T1)				M _2_ (Revenge T1)				*IV* (CA T2)		
Antecedent		B	SE (HCE3)	*p*		B	SE (HCE3)	*p*		B	SE (HCE3)	*p*
X (CV)	*a_1_*	0.58	0.07	<0.001	*a_2_*	0.31	0.09	<0.001	*c’*	0.29	0.04	<0.001
M_1_ (Stress)					*d_21_*	0.25	0.06	<0.001	*b_1_*	0.08	0.03	<0.01
M_2_ (Revenge)									*b* *_2_*	0.05	0.02	<0.01
Constant	*i_M1_*	−0.45	0.24	0.05	*i_M2_*	2.46	0.36	<0.001	*i_Y_*	−0.12	0.13	0.36
R^2^		0.14				0.06				0.16		
F (*df*)		45.438 *** (3, 975)				15.558 *** (4, 974)				12.754 *** (5, 973)		

Note: Bootstrap sample size = 10,000. Abbreviations CV = cybervictimization; IV = cyberbullying aggression; M = Mediator; SE(HC3) = Heteroscedasticity-Consistent Standard Error; T1 = Time 1; T2 = Time 2; *a*, *b*, *c*, *c*′, *d,* and *i* represent unstandardized regression coefficients. *** *p* < 0.001.

**Table 3:** Regression coefficients, standard errors, and model summary information for stress and avoidance as mediators.

	Consequent											
		M _1_ (Stress T1)				M _2_ (Revenge T1)				*IV* (CA T2)		
Antecedent		B	SE (HCE3)	*p*		B	SE (HCE3)	*p*		B	SE (HCE3)	*p*
X (CV)	*a_1_*	0.58	0.07	<0.001	*a_2_*	0.31	0.09	<0.001	*c’*	0.29	0.04	<0.001
M_1_ (Stress)					*d_21_*	0.25	0.06	<0.001	*b_1_*	0.08	0.03	<0.01
M_2_ (Revenge)									*b* *_2_*	0.05	0.02	<0.01
Constant	*i_M1_*	−0.45	0.24	0.05	*i_M2_*	2.46	0.36	<0.001	*i_Y_*	−0.12	0.13	0.36
R^2^		0.14				0.06				0.16		
F (*df*)		45.438 *** (3, 975)				15.558 *** (4, 974)				12.754 *** (5, 973)		

Note: Bootstrap sample size = 10,000. Abbreviations CV = cybervictimization; IV = cyberbullying aggression; M = Mediator; SE(HC3) = Heteroscedasticity-Consistent Standard Error; T1 = Time 1; T2 = Time 2; *a*, *b*, *c*, *c* ′, *d,* and *i* represent unstandardized regression coefficients. *** *p* < 0.001.
